# Simulating Local Deformations in the Human Cortex Due to Blood Flow-Induced Changes in Mechanical Tissue Properties: Impact on Functional Magnetic Resonance Imaging

**DOI:** 10.3389/fnins.2021.722366

**Published:** 2021-09-21

**Authors:** Mahsa Zoraghi, Nico Scherf, Carsten Jaeger, Ingolf Sack, Sebastian Hirsch, Stefan Hetzer, Nikolaus Weiskopf

**Affiliations:** ^1^Department of Neurophysics, Max Planck Institute for Human Cognitive and Brain Sciences, Leipzig, Germany; ^2^Methods and Development Group Neural Data Science and Statistical Computing, Max Planck Institute for Human Cognitive and Brain Sciences, Leipzig, Germany; ^3^Institute for Medical Informatics and Biometry, Carl Gustav Carus Faculty of Medicine, TU Dresden, Dresden, Germany; ^4^Department of Radiology, Charité – Universitätsmedizin Berlin, Berlin, Germany; ^5^Berlin Center for Advanced Neuroimaging, Charité – Universitätsmedizin Berlin, Berlin, Germany; ^6^Berlin Center for Computational Neuroscience, Berlin, Germany; ^7^Faculty of Physics and Earth Sciences, Felix Bloch Institute for Solid State Physics, Leipzig University, Leipzig, Germany

**Keywords:** biophysical modeling, simulation, tissue mechanics, blood flow, deformation, BOLD, VASO, fMRI

## Abstract

Investigating human brain tissue is challenging due to the complexity and the manifold interactions between structures across different scales. Increasing evidence suggests that brain function and microstructural features including biomechanical features are related. More importantly, the relationship between tissue mechanics and its influence on brain imaging results remains poorly understood. As an important example, the study of the brain tissue response to blood flow could have important theoretical and experimental consequences for functional magnetic resonance imaging (fMRI) at high spatial resolutions. Computational simulations, using realistic mechanical models can predict and characterize the brain tissue behavior and give us insights into the consequent potential biases or limitations of *in vivo*, high-resolution fMRI. In this manuscript, we used a two dimensional biomechanical simulation of an exemplary human gyrus to investigate the relationship between mechanical tissue properties and the respective changes induced by focal blood flow changes. The model is based on the changes in the brain’s stiffness and volume due to the vasodilation evoked by neural activity. Modeling an exemplary gyrus from a brain atlas we assessed the influence of different potential mechanisms: (i) a local increase in tissue stiffness (at the level of a single anatomical layer), (ii) an increase in local volume, and (iii) a combination of both effects. Our simulation results showed considerable tissue displacement because of these temporary changes in mechanical properties. We found that the local volume increase causes more deformation and consequently higher displacement of the gyrus. These displacements introduced considerable artifacts in our simulated fMRI measurements. Our results underline the necessity to consider and characterize the tissue displacement which could be responsible for fMRI artifacts.

## Introduction

Understanding the complex anatomical and functional architecture of the human brain has been a major research topic for more than a century. However, little is known about the dynamic interaction between mechanical tissue properties and the shape and microscopic composition of cortical substructures. For example, recent studies suggest that the gyrification of the developing brain can be explained by different growth rates of gray and white matter (Tallinen et al., 2016) that causes mechanical stress resulting in the characteristic pattern of gyri and sulci (Armstrong et al., 1995). This early stage of the nervous system’s development is followed by axonal growth patterns that are guided by mechanical cues, i.e., the tissue’s local stiffness gradients (Koser et al., 2016; Oliveri et al., 2021). Abnormalities in cortical folding, neuronal growth, and viscoelastic tissue properties have been associated with various neurological and cognitive disorders (Bayly et al., 2014) such as hydrocephalus, Alzheimer’s disease, multiple sclerosis, epilepsy, schizophrenia, autism, and brain tumors (Hardan et al., 2004; Lin et al., 2007; Wuerfel et al., 2010; Murphy et al., 2011; Streitberger et al., 2011, 2020; Li et al., 2016; Garcia et al., 2018b; Huesmann et al., 2020) which demonstrates a clear need for understanding the underlying tissue mechanics.

The rapid development of magnetic resonance imaging (MRI) methods has enabled the non-invasive observation of the complex structure-function relationship within the living brain at different timescales, i.e., by detecting morphometric or microstructural changes during development, disease, learning (Maguire et al., 2000; Draganski et al., 2004), or during rapid functional stimulation (Watanabe et al., 2017). Recent structural and functional MRI (fMRI) methods are approaching the spatial resolutions needed for resolving single cortical layers (Fukunaga et al., 2010; Trampel et al., 2019; Weiskopf et al., 2021) with a typical thickness around 400 μm (Wagstyl et al., 2020) that are specialized in processing specific neuronal information (Huber et al., 2017; Fracasso et al., 2018). The raised energy demand during elevated neuronal activity induces vasodilation in the activated brain tissue resulting in an increased inflow of freshly oxygenated blood. The primary fMRI contrast mechanisms rely on this vasodilatory response evoked by neuronal activity (Goense et al., 2016). In the context of high-resolution fMRI, the mechanical implications of vasomotion at the scale of gyral substructures have not yet been investigated (Hetzer et al., 2018a; Polimeni et al., 2018). It should be noted that minimal tissue displacements in the range of some percent of the fMRI voxel size can result in erroneous relative fMRI signal changes of the same magnitude, i.e., up to 10% for a 50-μm displacement within a 500 μm voxel at high contrast interfaces. For example, varying partial volume contributions of cerebrospinal fluid (CSF) can significantly impact the cortical fMRI signal due to its long transverse relaxation time as compared to cortical tissue (Piechnik et al., 2009). These mechanical effects are expected to depend on the baseline biomechanical properties of the tissue and may thus depend on pathologies accompanied by tissue degeneration like protein deposition, dystrophic calcification, gliosis, etc. (Yin et al., 2018).

To our knowledge, the extent to which the fMRI signal is influenced by tissue deformations inherent to the mechanical behavior of cortical brain tissue has never been systematically investigated. Therefore, we here combine simulations of tissue biomechanics and the physics of fMRI to study whether subtle mechanical property changes due to vasodilation can result in artifacts in ultra-high resolution fMRI. Two effects of vasodilation on tissue mechanics will be explored.

First, the viscoelastic properties locally change due to vasodilation (Parker, 2017). Recent studies using MR elastography (MRE) (Muthupillai et al., 1995) detected slight changes in viscoelastic properties of human brain tissue during hypercapnia-induced vasodilation (Hetzer et al., 2018b). This is partially predicted by the well-known Fåhræus–Lindqvist effect (Fåhræus and Lindqvist, 1931), which describes the increase of blood viscosity with vessel diameter. Embedded in folded cortical tissue that is under stress (Xu et al., 2010; Tallinen et al., 2016), a focal increase of tissue stiffness due to vasodilation will result in slight displacements of the mechanically interconnected surrounding tissue.

Second, a focal volume change is expected. Within a constant intracranial volume as assumed in the Monro–Kellie doctrine, any variation in cerebral blood volume (CBV) must be compensated by opposite changes in other compartments, or must affect the intracranial pressure (ICP). While clinically induced vasoconstriction is routinely used to reduce ICP in patients with severe head injury (Laffey and Kavanagh, 2002), the mechanical effects of a focal vasodilatory volume increase of activated tissue and the associated displacement of interconnected cortical tissue is expected to be fully compensated by CSF redistributions without significantly elevating the ICP in accordance with the Monro–Kellie doctrine (Piechnik et al., 2009; Jin and Kim, 2010). With a typically observed relative blood volume increase of 50% in proximity of neuronal activation (Lu et al., 2003) the increase of the resting cortical blood partition fraction of 5% (Hua et al., 2011) is expected to translate into a local tissue volume increase of a couple of percent and concomitant shifts.

To estimate the tissue displacements due to vasodilation, we created a two-dimensional geometric model of a representative gyrus from ultra-high resolution histology data of Amunts et al. (2013) and used a finite-element model to simulate the respective tissue mechanics. Following recent works realistically modeling the nearly incompressible brain tissue (Franceschini et al., 2006; Bayly et al., 2013; Budday et al., 2014) our model includes neo-Hookean material properties for white matter (WM) and cortical gray matter (GM) resembling six neocortical layers. For simplicity, neither CSF compartment changes, CSF redistributions due to focal deformations of cortical tissue nor gravitational forces were explicitly modeled as the brain suspended in CSF exists in neutral buoyancy. A quasi-static simulation with hyperelastic material was chosen as an appropriate model for slow deformations or the equilibrium state.

To predict the sensitivity of typical high-resolution fMRI sequences to the analyzed biomechanical effects, we simulated the MR imaging process at different image resolutions on an idealized 7-Tesla scanner with a high-performance gradient system which will be realistically available within the next years and used to study the intracortical functional neuroanatomy (Brain Initiative, 2021).

## Materials and Methods

### Anatomical Model

**FIGURE 1:**
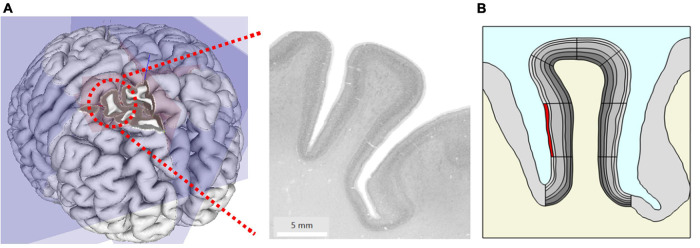
**(A)** Original microscopy image of the right middle frontal gyrus at MNI coordinate *x* = 24 mm, *y* = 30 mm, *z* = 41 mm taken from the HumanBrainProject. **(B)** The geometric model extracted from **(A)** showing gray matter (GM) (gray) with different anatomic layers (different gray levels), white matter (WM) (cream) and cerebrospinal fluid (CSF) (blue). Simulation of local changes in tissue mechanics: the stiffness and volume of a segment of interest (here superficial layer L1 highlighted in red) were increased to simulate the effect of local increase in blood flow.

From ultra-high resolution histology data at nearly cellular resolution of 20 μm from the HumanBrainProject (Amunts et al., 2013), we created a two-dimensional geometric model of a coronal slice of the right middle frontal gyrus (at MNI coordinate 24, 30, and 41) of 8.5 mm width and 15.3 mm length (Figures 1A,B).

We traced the cortical boundaries of the gyrus in the histological image and imported the boundaries for subsequent finite element simulations in COMSOL Multiphysics (V.5.5, COMSOL Multiphysics Göttingen). We modeled the geometry with an adaptive triangular mesh with elements size ranging from 0.38 to 190 μm. To ensure high numerical accuracy, the extremely fine mesh size was used for the cortical gyrus region in our study. The six cortical layers in GM were manually traced by an expert with more than 10 years of experience in histology and neuroanatomy (CJ) and later incorporated into the model. The geometry also includes the surrounding GM, WM, and CSF. Note that we did not explicitly model the biomechanics of CSF in this study. To separately specify parameters locally in different parts of the layered geometry during simulation and analysis, we divided the model further into segments (Figure 1B).

To assess potential simulation resolution effects, the simulations were repeated using different mesh element sizes from normal to extremely fine. The mesh size did not influence our results much: for fine with average mesh element size of 206 μm and extremely fine mesh with average mesh element size of 143 μm, the difference in resulting displacement was less than 0.05% (20 nm) indicating that convergence had been attained.

### Biomechanical Simulations

We modeled the brain tissue as an isotropic and hyperelastic material as suggested in Xu et al. (2010) and Tallinen et al. (2016). We assumed a quasi-static regime disregarding any dynamic effects, such as viscosity or hydrodynamic interactions between parenchyma and CSF. Accounting for dynamic effects would dramatically increase the complexity of the physical model and the number of coupling parameters and is therefore left for future studies. We use the strain-energy density function *W* of a nearly incompressible neo-Hookean material (Holzapfel, 2000) defined as


$$W=\frac{\mu }{2}\,\left[\text{tr}\,\left({\text{FF}}^{T}\right)\,J^{-\frac{2}{3}}-3\right]+\frac{\kappa }{2}\,{\left(J-1\right)}^{2}$$

with μ and κ being the shear and bulk modulus of the material, F the deformation gradient and


J=det⁢F

Using this, the constitutive stress-strain relation is given by


$$\sigma =\frac{1}{J}\,\frac{\partial W}{\partial \text{F}}\,{\text{F}}^{T}$$

where nearly incompressible tissue properties are achieved when κ≫μ.

The cortical gyrus is modeled and simulated in COMSOL for characterizing blood flow-induced motion and volume change by means of biomechanical tissue properties. Its material properties were taken from Budday et al. (2015) and Mcintosh and Anderson (2010) for the GM with a shear modulus of 1.4 kPa, approx. 35% stiffer WM (1.9 kPa) and assuming a nearly incompressible material with κ three orders of magnitude larger than μ (Xu et al., 2010). An overview of the specific values used for simulation are given in Table 1.

**TABLE 1:** An overview of the material parameters used for mechanical simulation.

	**Gray matter**	**White matter**
μ (Pa)	1,400	1,900
κ (GPa)	1.4	1.9
ρ (kg m^–3^)	1,040	1,040

In order to gauge the effect sizes and contributions of different tissue characteristics to the fMRI signal, we studied three computational models simulating different biomechanical mechanisms: stiffness change, volume change, and a combination of both. Based on recent research using direct measurements, we know that there is tension in WM and compressive stresses in cortical GM in both smooth mouse brain and folded ferret brain (Xu et al., 2009, 2010; Garcia et al., 2018a). This implies that the brain scanned by *in vivo* MRI methods is under stress already. We estimated the compressive stress distribution in the gyrus based on the assumption that flattening the folded cortex results in a realistic stress distribution. The underlying simplifying assumption is that the folding pattern results from simple mechanical effects without tissue growth or other influences. In order to obtain the flattened geometry, the input geometry was deformed back to a smooth and unfolded shape. The inner (lower) and outer (upper) gyrus boundaries were subjected to a prescribed displacement in y direction (top-down direction on Figure 1B); it means they were straightened into two parallel lines with a given distance. This was done in small steps in which the gyrus became more and more deformed until these boundaries were flat and even (see Supplementary Video 1). During this procedure, the stress inside the gyrus, especially in curved regions, would increase as well as the overall length, as one would expect. The stresses arising from this deformation were mapped back to the original gyrus shape. The resulting stress distribution was mapped onto the folded input geometry and used for the starting configuration for the simulation studies involving stiffness change.

As a first computational model, solely stiffness changes were taken into account. Then, only focal volume changes were considered. Finally, we combined both volume and stiffness changes in our third model. The following steps and parameters were used for these three simulations:

1. To simulate the effect of stiffness change, we locally increased the shear modulus μ_1_ of one specific segment of a single layer (highlighted in red in Figure 1B) by up to 10% of its initial value μ_0_, i.e., μ_1_ = 1.1 μ_0_. To trace the dynamics of the resulting shape deformation, we swept the stiffness value from μ_0_ to μ_1_ in equidistant steps of 0.5%.
2. The same segment as used in the previous model (Figure 1B) underwent a volume increase by 10%. The volume change occurred along two orthogonal directions in plane (i.e., the extent in the through plane direction was implicitly kept constant). The volume increase was governed by expanding the lateral dimension uniformly in equal steps of 0.5%.
3. In the final model, both volume and stiffness change were combined in an individual simulation. The two parameters were changed simultaneously up to 10%.

To assess the sensitivity of our model on the chosen parameter values and to reasonably determine the tissue mechanical properties, we set up a grid simulation that captures a large range of the parameters κ and μ obtained from literature (Budday et al., 2016, 2020; Ganpule et al., 2018).

For quantifying the resulting tissue displacements, a displacement field with the coordinates *u*, *v* was defined. At a given coordinate (X, Y), e.g., after deformation of the geometry, the values *u* and *v* quantify the displacement in x- and y-direction, respectively, relative to its original position. The total displacement was then defined as$d_{t\,o\,t}=\sqrt{u^{2}+v^{2}}$.

### Functional Magnetic Resonance Imaging Simulations

To predict the sensitivity of different typical high-resolution fMRI acquisitions to biomechanical tissue displacements due to vasodilatory effects, the gyral geometries and their changes resulting from the mechanical simulations were transferred to MRiLab,^1^ an open-source software simulating the entire MR imaging process (Liu et al., 2017).

The three most widely used fMRI variants (Goense et al., 2016; Huber et al., 2019) were simulated, i.e., Vascular-Space-Occupancy (VASO), blood-oxygen-level-dependent (BOLD), and proton density contrast as used in arterial spin labeling (ASL), for the next generation 7-Tesla scanner with a high-performance gradient system (maximal gradient amplitude and slew rate: 200 mT/m, 1,000 T/m/s), which is tailored for studies of the mesoscopic cortical functional neuroanatomy, and idealized radio frequency (RF) coils and B_0_ homogeneity employing a gradient-echo readout with optional inversion recovery preparation.

Proton density values and relaxation times at 7 Tesla for CSF, WM and cortical layers (see also Table 2) were taken from the literature (Rooney et al., 2007; Tofts, 2010; Carey et al., 2018; Caan et al., 2019; McColgan et al., 2021) and mapped to the mechanical gyrus model.

**TABLE 2:** Values for proton density (PD), T1, T2* for different tissue types and cortical layers (L1–6) at 7 Tesla used in the simulations.

	**PD (%)**	**T1 (ms)**	T2* (ms)
CSF	100	4,400	600
L1	86	2,200	38
L2	85	2,080	36
L3	83	1,960	34
L4	81	1,840	32
L5	79	1,720	30
L6	78	1,600	28
WM	69	1,200	24
GM	82	1,900	33

For gyral geometries resulting from the analyzed focal stiffness and volume changes, magnitude MR images were reconstructed from the simulated k-space data at different isotropic image resolutions (voxel sizes of 0.25, 0.5, and 1.0 mm) of the following fMRI sequence types:

(a) Proton density contrast was created at a short echo time *TE* = 5 ms to reflect the effort of blood flow measurements to suppress unwanted concomitant BOLD signal, i.e., in ASL (Hetzer et al., 2011; Huber et al., 2019).
(b) BOLD imaging was simulated by calculating the image contrast at *TE* = T2^∗^ of GM where BOLD sensitivity is optimal (Deichmann et al., 2002).
(c) VASO contrast (*TE* = 5 ms) was simulated by an inversion recovery preparation module for nulling blood with T1b = 2,100 ms (Zhang et al., 2013) at an inversion time of *TI* = ln(2)^∗^T1b = 1,456 ms.

Voxel-wise maps of the relative signal change resulting from tissue displacement were calculated from the corresponding MR images of the modeled gyrus in the resting and “activated” conditions, i.e., by subtracting the rest from the activated condition image.

## Results

### Mechanical Simulations

#### Cortical Stress Distribution

**FIGURE 2:**
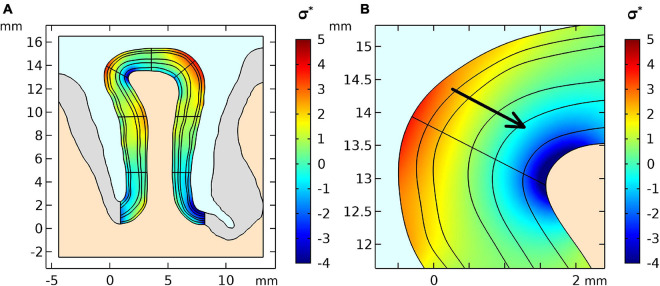
**(A)** Stress distribution results of the first simulation step of the geometry and **(B)** a zoomed region where positive curvature results in a positive stress gradient across layers. The stress was normalized by the stiffness value μ of the GM.

From our biomechanical simulation employing the flattening approach, we obtained an estimate of the stress distribution shown in Figure 2. The stress distribution indicates parts with negative stress which correspond to compression in high curvatures and positive stress distribution which corresponds to tension in low curvatures. The arrow shows the stress gradient from highest to lowest stress value.

#### Stiffness Change

**FIGURE 3:**
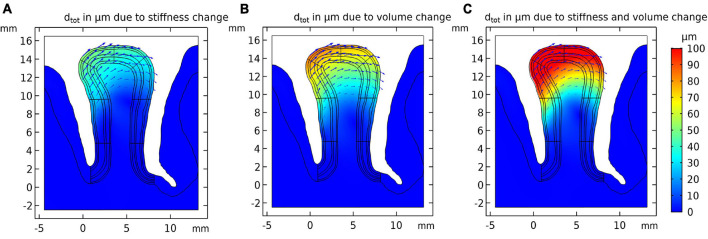
Resulting tissue movement for a 10% increase of **(A)** tissue stiffness, **(B)** tissue volume of the activated layer segment, and **(C)** the combination of both effects. Displacement fields are shown by black arrows and color scale indicates magnitude of displacement.

Using the model set up in “Cortical Stress Distribution,” we conducted a simulation study to estimate the effect of local stiffness change (e.g., due to blood flow) and the resulting deformation of the cortical tissue. Figure 3A shows the simulation results. One can observe that the largest displacement took place in the gyrus crown, and the displacement decreased with the distance from the gyrus peak. The displacement of the gyrus correlated with the amount of stiffness change. We observed a maximum total displacement of 41.3 μm for the gyrus crown with a 10% stiffness change.

#### Volume Change

The displacement pattern arising from a local volume increase of the activated gyral segment was similar to the displacements induced by a local stiffness increase (Figure 3B), with an estimated 69.5 μm total displacement for a volume increase of 10% in the gyral crown. This effect was hence larger than the local stiffness change (Figure 3A).

#### Combined Stiffness and Volume Change

Figure 3C shows the results for the combined effect of simultaneous increase in stiffness and volume by 10% each, resulting in a larger displacement compared to the individual effects. The total displacement calculated in the gyrus crown was 110.7 μm.

**FIGURE 4:**
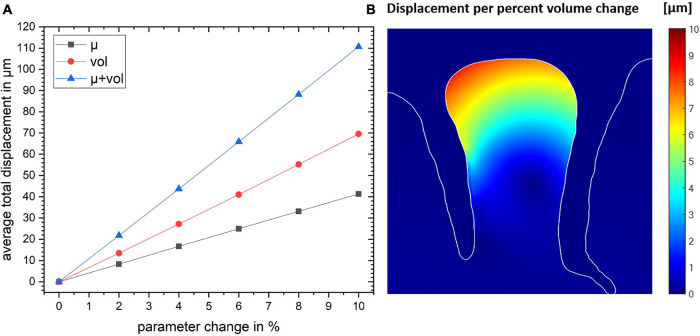
**(A)** The total displacement for the gyrus crown as a function of parameter change for the three simulation studies. **(B)** The spatial map of the resulting displacement given in per percent volume change. Color map shows the magnitude of local displacement.

The total displacement of all three models as a function of the parameter change is shown in Figure 4. The plot shows that the total displacement increases linearly with increasing stiffness or volume change with the local volume increase resulting in a larger displacement. Our simulations show that the combination of these two mechanisms are additive (to within an accuracy of <0.1 μm).

#### Robustness of the Simulation and Sensitivity to Tissue Parameter Choices

**FIGURE 5:**
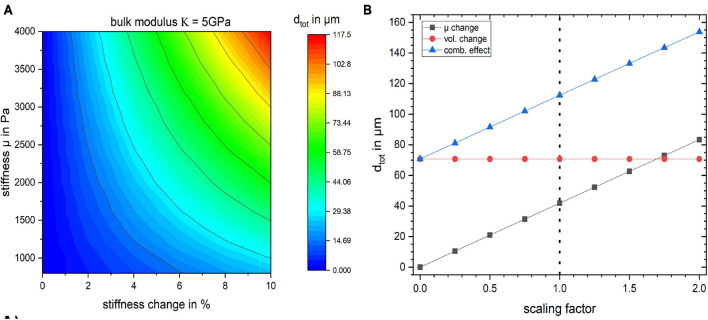
**(A)** Surface plot of the total displacement for the studied region as a function of baseline stiffness and stiffness change in percent for bulk modulus κ = 5 GPa. The baseline stiffness μ = 1,400 Pa is used for all the previously discussed studies. **(B)** The effect of scaling the stress field on tissue displacement for the three simulation cases. The dotted line indicates the stress resulting from the flattening procedure employed in this work.

We conducted a simulation in which we investigated a broader range of mechanical parameters summarized in Table 3. The results are shown in Figure 5 and Supplementary Figure 2. The stiffness change was kept constant (2 and 10%, respectively) and the total displacement *d*_*tot*_ as a function of the bulk modulus κ and the baseline stiffness μ of the gyrus was calculated (Supplementary Figure 1). The values for *d*_*tot*_ ranged from 4.69 to 23.90 μm for a change in stiffness of 2% and from 23.00 to 117.50 μm for a stiffness change of 10%.

**TABLE 3:** Parameter range for mechanical properties based on the literature (Xu et al., 2010; Budday et al., 2020).

	**Gray matter**	**White matter**
μ (Pa)	800–4,000	1,120–5,600
κ (GPa)	0.5–5	0.5–5

The influence of κ on the displacement of the gyrus is negligible since the material model is nearly incompressible. In Figure 5 the bulk modulus was kept at a fixed value of κ = 5 GPa and *d*_*tot*_ of the gyrus as a function of its stiffness value μ and its stiffness change (in %) is shown.

Furthermore, in order to gauge how the baseline stress distribution impacts the results, the simulations were repeated with different scales of obtained stress distribution from the flattening step. The results in Figure 5B show that the displacement values depend on the global scaling of the stress distribution. The dotted line shows the scaling factor of 1 which was used for all other simulations. The internal stress distribution was not relevant for the volume change simulation.

**FIGURE 6:**
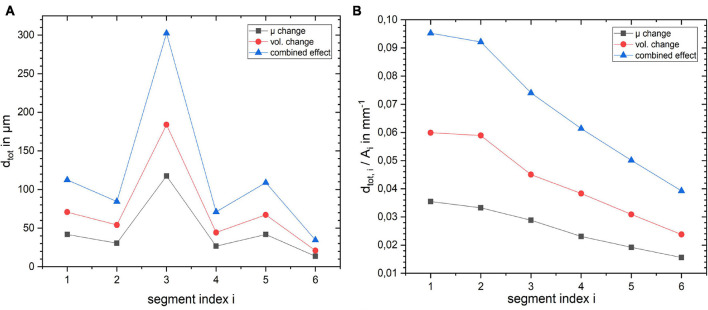
Influence of layer position within the cortical segment on **(A)** absolute displacement for the three different scenarios and **(B)** displacement normalized by the layer segment volume.

To get a deeper understanding of how the layer depth influences the biomechanical response of the cortex, we repeated the simulations for the same lateral segment across all layer depths (outermost layer *i* = 1 to innermost layer *i* = 6). The results for a change in stiffness μ, volume and the combined effect by 10% are shown in Figure 6. Figure 6A shows how *d*_*tot*_ changes with increasing index *i*, i.e., outer to inner layer, when changing the segment from outer to inner layer. One can observe that the larger displacement occurred in layer 3 for all three cases, as expected with the maximum for the combined effect. To rule out the effect of the segment size we normalized the resulting *d*_*tot*_ of a segment *i* by its area *A*_*i*_ as shown in Figure 6B. The total displacement *d*_*tot*_ per area *A*_*i*_ clearly decreases with increasing segment index *i*, with a factor of more than 2 between the innermost (*i* = 6) and outermost (*i* = 1) layer. This effect was largest for the combined stiffness and volume change.

To investigate how the effects depend on the size of the region experiencing blood flow changes, we extended the simulation region from one segment to four. In all three scenarios, the extended activation of segments resulted in a larger displacement. For example, when the activated segment was extended up to layer 4, the total displacement for volume increase simulation increased from 69.5 to 319.7 μm. Also, when the position of the broad simulated segments was moved to other regions, it resulted in larger displacements compared to a single segment.

Changing the position of the activated segment for the first layer in tangential direction, the total displacement in the gyrus crown decreased for all the three cases (Supplementary Figure 3A). By extending the length of the activated region from a single segment to four segments, we observed an increase in the displacement value for all the three simulation cases. For example the displacement value for segment one of layer one for stiffness change simulation was 29.9 μm. However, the displacement value increased as we increased the segment number, reaching 74.4 μm (Supplementary Figure 3B).

### Spurious Functional Magnetic Resonance Imaging Signal Changes Due to Displacement

**FIGURE 7:**
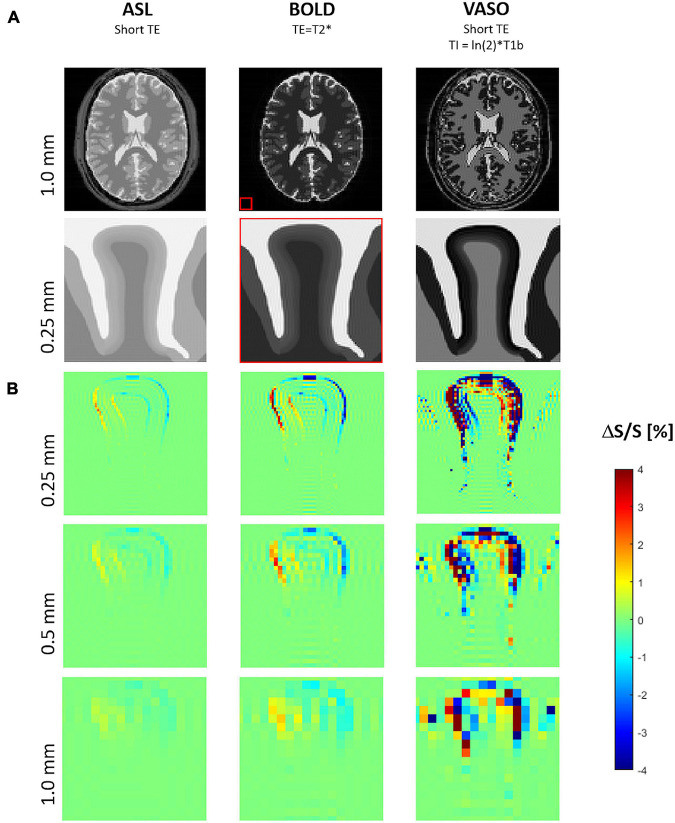
**(A)** Simulated MR images for three typical functional magnetic resonance imaging (fMRI) sequence types with proton density contrast (e.g., ASL), BOLD contrast, VASO contrast. The biomechanically modeled gyrus is shown below the corresponding image of a digital brain phantom of MRiLab for illustrating the typical whole-brain MR contrasts seen at 7 Tesla and the relative spatial dimensions (red box). **(B)** Maps of signal change due to tissue displacement following a 2% increase of both volume and stiffness of the activated layer segment (see also Figure 1B) at three different resolutions, i.e., voxel sizes of 1, 0.5, and 0.25 mm.

Raw images of the gyrus in the resting condition for the three analyzed fMRI sequence variants at three image resolutions are shown in Figure 7A. The corresponding maps of spurious fMRI signal changes due to a tissue displacement following a 2% volume increase of the “active” layer segment (marked red in Figure 1B) can be seen in Figure 7B.

Signal changes due to tissue displacement were strongest in fMRI methods based on images with high contrast between neighboring tissues and cortical layers, i.e., with effect sizes decreasing from VASO to BOLD and proton density contrast (e.g., ASL). Further, the artifacts due to displacements increased with the image resolution.

For all fMRI variants, the shift of gyral tissue from left to right (Figure 3) resulted in strongest signal changes along the pial surface, i.e., the boundary of cortical tissue to CSF, with a signal increase on the left side and a decrease of signal on its right side. Similar effects of smaller magnitude can be seen along the boundary between the cortex and WM – with inverted sign in the case of VASO fMRI.

Note the typical signal oscillations of the Gibb’s ringing artifacts (Kellner et al., 2016) unfolding orthogonally to the CSF/GM border within gyral tissue and CSF.

## Discussion

Our study addressed the impact of mechanical effects of blood flow/volume changes on the spatial resolution and artifacts in fMRI by mechanical modeling paired with MRI signal models. We generated a mechanical tissue model to estimate the range of mechanical displacement that is expected in cortical layers of the human brain. This model will be briefly discussed in the following paragraph.

### Mechanical Simulations

Since there are not yet accurate methods to estimate the exact values of stress within brain tissue *in vivo*, we used the geometry itself as the input variable. The shape of the tissue is a result of all forces acting on the brain during development and we regard the tissue as a confined geometry subjected to mechanical stresses during development. We estimate these stresses by numerically inverting the developmental process and bringing the geometry to a smooth, unfolded shape. This stage is typically considered as the starting point for established models of cortical folding (Bayly et al., 2013; Tallinen et al., 2016). Our simulation of the initial stress distribution for the cortical gyrus (Figure 2) shows a good agreement with the results presented by other studies [See Figure 7 in Xu et al. (2010)], which use the forward growth model by Rodriguez et al. (1994). Based on this initial stress distribution and assuming a local change of tissue stiffness due to blood flow, our simulations predict a considerable displacement of the tissue in the gyral crown of 4 μm per percent stiffness change. Independently of the initial distribution of stresses in the tissue, our results show that local volume changes also induce tissue displacements. Here, the effect would even be higher leading to a displacement of 7 μm per percent volume change in our simulation. Our study indicates that the combination of the two different effects, i.e., displacements due to local stiffness and volume change are cumulative. Overall, our simulations predict a linear increase in displacement with increasing local stiffness or volume. This sets a lower bound to the expected effect in real experimental settings, since our presented results are based on conservative estimates of parameters as the values are taken from *ex-vivo* measurements for tissue stiffness via indentation, which underestimate stiffness of perfused tissue. Furthermore, the volume and stiffness changes from literature were likely underestimated due to partial volume effects of low-resolution data.

### Functional Magnetic Resonance Imaging Simulations

Our fMRI simulations show that the predicted tissue displacements following focal vasodilatory activity in a gyrus can create erroneous fMRI signal changes on the same order of magnitude as typically reported for classical fMRI contrast mechanisms, i.e., between 0.5 and 5% signal change of the raw images acquired in VASO, BOLD, or ASL fMRI (Huber et al., 2019). Both positive and negative signal changes resulting from these anatomical deformations will interfere with the true fMRI signals of interest that are more directly coupled to neuronal activity reflecting excitatory or inhibitory neural processes. Further, the spatial precision of fMRI may be significantly impacted. The peak of these erroneous signal changes can be located some centimeters away from the origin of the focally activated capillary bed embedding the neuronal activity, i.e., from the base to the crown of the exemplary gyral structure modeled in our simulations.

Importantly, spurious signal changes due to activity-induced tissue displacements are strongest in fMRI methods based on images with high contrast between neighboring tissues, i.e., with effect sizes considerably decreasing from VASO to BOLD and proton density contrast (e.g., ASL). Therefore, the relative fMRI sensitivity of ASL compared to VASO might be empirically underestimated – especially at high-resolution fMRI. Further, it should be noted that fMRI variants nulling signal of specific tissue compartments like VASO or comparable methods (Shen et al., 2009) can result in erroneously high relative signal changes in those voxels (division by zero) and should therefore be interpreted with highest care. For example, cortical layers with T1 values matching T1b will be nulled in VASO fMRI and therefore create singularities in the relative signal change map.

As expected, the displacement fMRI signal change increases with the image resolution due to partial volume effect, i.e., following the ratio of the tissue displacement D and the voxel size V. Generally, the relative signal change due to tissue displacement can be approximated by Δ*S*/*S* = *C* × *D*/*V* with the relative tissue contrast *C*. In the example of idealized proton density contrast (with the lowest artifact strength compared to BOLD or VASO), the maximum tissue contrast *C* is found on the pial surface (creating a sharp discontinuity of proton density, i.e., 80% for GM and 100% for CSF). Assuming a tissue displacement of only 0.02 mm (∼double of a capillary diameter) resulting from 2% focal volume and stiffness changes (the lower limit of the explored range herein) predicts signal changes of 2, 1, and 0.5% with isotropic voxel sizes of 0.25, 0.5, and 1.0 mm, which is in good agreement with the corresponding signal change maps of our fMRI simulations.

For simplicity we assumed a linear increase of T2^∗^ values across cortical layers going from the white interface to the pial surface. Different T2^∗^ distributions across layers would lead to slightly different results. A more complex laminar T2^∗^ distribution, e.g., the one observed by McColgan et al. (2021) with a rapid drop of T2^∗^ in the outermost layer likely due to superficial veins, leads to a contrast increase between CSF and superficial GM. This would further slightly amplify the effects predicted by our simulations, especially in fMRI sequences employing longer echo times like BOLD fMRI.

Generally, resolving cortical layers and detecting the predicted tissue shift artifacts requires very high spatial resolution enabled by higher magnetic field strength and the associated increase of signal-to-noise ratio. However, at a given resolution the tissue shift effect is not expected to depend strongly on field strengths (in the range commonly available in human or animal MRI). Independent of the T1 relaxation of brain tissue at different field strengths, the GM-CSF contrast that dominates the simulated effect will peak for VASO sequences suppressing the signal of GM tissue. In BOLD fMRI, a potential decrease of GM-CSF contrast due to slower T2^∗^ relaxation at lower field will generally be compensated by the choice of *TE* = T2^∗^ of GM which provides optimal BOLD fMRI sensitivity. However, the significant decrease of R2^∗^ of pial veins at lower field strengths (Gati et al., 1997) will slightly dampen the tissue shift effect for BOLD fMRI at lower field. Independent of field strength, the tissue shift effect is minimal for ASL sequences at short *TE* due to the relatively small proton density difference between CSF and brain tissue. In line with the presumed robustness of the tissue shift effect across static magnetic field strengths it might contribute to functional signal changes observed in multi-echo BOLD fMRI at very short echo times independently from the main magnetic field strength (Markuerkiaga et al., 2021).

The conservative assumptions used in our biomechanical simulations tend to underestimate activity-induced tissue displacements. Based on our analyses, different choices of the relevant mechanical parameters translate linearly (within the ranges explored) into tissue displacements and the associated erroneous fMRI signal changes, respectively. Representative parameters are the already discussed baseline stiffness of cortical tissue (taken from *ex-vivo* data) and the GM stiffness increase of 2% taken from hypercapnia MRE experiments acquired with 2.0 mm isotropic voxels (Hetzer et al., 2018b) barely matching the thickness of cortical GM (Fischl and Dale, 2000). Assuming that the stiffness increase following vasodilation is driven by the highly vascularized layers only, i.e., 1/3 of the voxel volume, a three times higher stiffness change in the activated layer segment might be more realistic (partial volume effect) translating into three times higher signal changes due to the increased tissue displacement.

Further, higher erroneous displacement fMRI signal changes can be expected following our observation that the displacement scales with the activated volume. Our choice of the thin layer 1 to be activated in our fMRI simulation falls within the lower limit of displacements of gyral tissue following neuronal stimulation. For example, we found a factor of 3 higher displacement when activating one central layer instead of layer 1. As neuronal activity is usually distributed across several layers in most fMRI paradigms, the activated tissue volume will probably be even higher while the highly vascularized and perfused central layers are expected to dominate local tissue displacement effects (Shen et al., 2008; Jung et al., 2021). We found that the relative displacement effect (normalized by the volume of the activated layer segment) slightly decreases from outer to inner layers. It should be noted that the influence of layer thickness variability across cortical GM on the tissue shift effect dominates the influence of the baseline stiffness variability (see also Figure 6) of roughly 10% across layers (Iwashita et al., 2014).

Furthermore, depending on the gyral geometry and the position of the activated layer segment therein, the resulting forces can also be effectively absorbed by the surrounding tissue without causing significant deformations. Such favorable neuroanatomical configurations, i.e., in functional areas with low gyrification or flat and thin gyri (Alemán-Gómez et al., 2013), might constitute a precondition for current state-of-the-art fMRI studies successfully showing high layer-specificity (Huber et al., 2018; Yu et al., 2019). Furthermore, with current layer fMRI techniques, it cannot be excluded that other noise sources (i.e., motion resulting from breathing and cardiac pulsations, or manual steps during layer segmentation) dominate and obscure the small functional tissue shift effects predicted in our work, especially as motion correction or pseudo-automatic layer segmentation are commonly focused to specific areas of interest (Huber et al., 2020).

Assuming that practical solutions are found for removing the harmonic non-rigid motion of brain tissue during the cardiac cycle (Terem et al., 2018; Adams et al., 2019), e.g., by triggering the MR acquisition, our simulations predict that future technical developments of high-resolution fMRI might have to consider local cortical deformations of tissue mechanically interconnected to the activated area. For example, automated dynamic layer segmentation methods capable of dissociating local deformations from intensity changes within activated layers might be needed to correct for local tissue shifts in order to detect fMRI signal changes more directly coupled to neuronal activity. Further, our simulations suggest that such approaches should also actively address Gibb’s ringing (Kellner et al., 2016) (creating alternating signal orthogonally to sharp image boundaries similar to layers) which will be amplified by the subtractive nature of fMRI in the context of tissue displacements.

We are not aware of any results in published layer fMRI studies directly corroborating the predicted gyral deformation effects, but this may be explained by them focusing on rather small regions of interest and applying artifact suppression methods. To address this question, a systematic meta-analysis of mesoscopic fMRI data may be conducted to detect potential inverted signal changes on the opposite sites of activated gyri as predicted by our simulations.

As tissue displacement effects are expected to unfold within the vasodilatory reaction time of some seconds following neuronal activity, fMRI with high spatiotemporal resolution will gain from addressing tissue displacement effects to better dissociate early neuronal-driven responses (i.e., rapid changes of blood relaxation time reflecting oxygen extraction) and the delayed vascular-driven responses (Jung et al., 2021). Models of the laminar BOLD response (Havlicek and Uludaǧ, 2020; Uludag and Havlicek, 2021) incorporating the effects of gyral displacements may not only sharpen the spatial precision of laminar fMRI (Fracasso et al., 2021) but also help interpreting fMRI signal changes associated with non-vascular mechanisms (Le Bihan et al., 2006; Roth and Basser, 2009; Patz et al., 2019).

### Limitations

Our simulation study delivers new insights into blood flow-induced local displacement of human brain tissue *in vivo*. However, there are some limitations: In our current proof-of-principle study, we restrict our simulation to a 2-dimensional model of one representative gyrus. Further research implementing a computationally demanding full 3D model of the brain with different activated gyri may shed further light on how our predicted effect sizes depend on the anatomical variability. The deformative effects predicted by our quasi-static model might be dampened or temporally shifted relative to the hemodynamic response depending on the unknown underlying CSF redistribution mechanism (Jin and Kim, 2010). As long as the physical origin and the laminar extension of the observed volume fraction change due to neuronal activity is not resolved, a computationally highly demanding dynamic viscoelastic model of the brain would have to include hydrodynamics and a high number of unknown parameters, i.e., friction, pressure differentials, physical volume exchange rates within tissue, or the dynamic vessel dilation process across layers, etc. The maps used by Jin et al. acquired at mesoscopic resolution would be a perfect candidate for future empirical studies exploring the sources of CSF redistributions caused by neuronal activity. Furthermore, the baseline tissue stiffness values were taken from the *ex-vivo* studies using indentation methods that might not exactly reflect *in vivo* values because brain properties change post-mortem (Bertalan et al., 2020). However, current *in vivo* MRE is limited in detecting cortical stiffness due to boundary effects on propagating shear waves (Hirsch et al., 2017). Moreover, we have neglected the impact of biomechanics of CSF in our simulation as well as the pia mater due to its thinness which is a few micrometers and much thinner than a cortical layer. Including the mechanical properties of CSF might slightly change the displacement field of cortical tissue. More interestingly, the CSF redistribution flow patterns resulting from gyral displacements could induce flow artifacts in fMRI signals (Duyn et al., 1994; Fultz et al., 2019) acquired near the pial surface where the highest CSF flow velocities are expected. In a future study we will include the hydrodynamics of this CSF redistribution in our simulations to analyze associated artifactual signal changes in high-resolution fMRI.

The study focused on biomechanics and did not address the myriad factors impacting fMRI measurements. Established BOLD-fMRI models show the importance of the interaction of blood flow, volume and oxygenation. They are increasingly refined to include the intricacies of the vascular system of the cortex including draining vein and orientation effects (Viessmann et al., 2019). Also the regulation of the blood flow remains an intense area of research on coupling of blood flow and different types of neuroelectric activity (Logothetis et al., 2001; Havlicek and Uludaǧ, 2020).

### Conclusion

In this biomechanical simulation study, we predict that increasing the resolution of fMRI to resolve single cortical layers is hindered by undesired small local deformations of cortical tissue mechanically interconnected to the activated area. Following the vasodilatory response, volume and stiffness changes of the activated layer lead to displacements realistically varying from 10 to 100 μm in gyral structures. The artifacts and erroneous changes in the predicted fMRI signal reached up to 5% – a similar magnitude as typical neurovascular responses. The artifactual signal changes increase with decreasing voxel size and are strongest in fMRI methods based on images with high contrast between neighboring tissues, i.e., with effect sizes considerably decreasing from VASO to BOLD and ASL fMRI. Although our simulations require experimental validation and would benefit from biomechanical properties measured *in vivo*, they suggest that focal biomechanical changes may relevantly affect ultra-high resolution fMRI experiments and deserve further consideration.

## Data Availability Statement

The original contributions presented in the study are included in the article/Supplementary Material, further inquiries can be directed to the corresponding author.

## Author Contributions

MZ and SHe contributed to the conception and design of the study, performed the simulations and the statistical data analysis, and wrote the first draft of the manuscript. MZ, SHe, NS, CJ, IS, SHi, and NW contributed substantially to interpretation of the data, revised the manuscript critically for intellectual content and have approved the submitted version.

## Conflict of Interest

The authors declare that the research was conducted in the absence of any commercial or financial relationships that could be construed as a potential conflict of interest.

## Publisher’s Note

All claims expressed in this article are solely those of the authors and do not necessarily represent those of their affiliated organizations, or those of the publisher, the editors and the reviewers. Any product that may be evaluated in this article, or claim that may be made by its manufacturer, is not guaranteed or endorsed by the publisher.

## Footnotes

1
http://mrilab.sourceforge.net
