# Supplementary material for: Simulating Local Deformations in the Human Cortex Due to Blood Flow-Induced Changes in Mechanical Tissue Properties: Impact on Functional Magnetic Resonance Imaging
Source: Front Neurosci. 2021 Sep 21;15:722366. doi: 10.3389/fnins.2021.722366 (PMC8490675; doi:10.3389/fnins.2021.722366)
Supplement: Supplementary file 2 [file Presentation_1.pptx]

## Slide 1
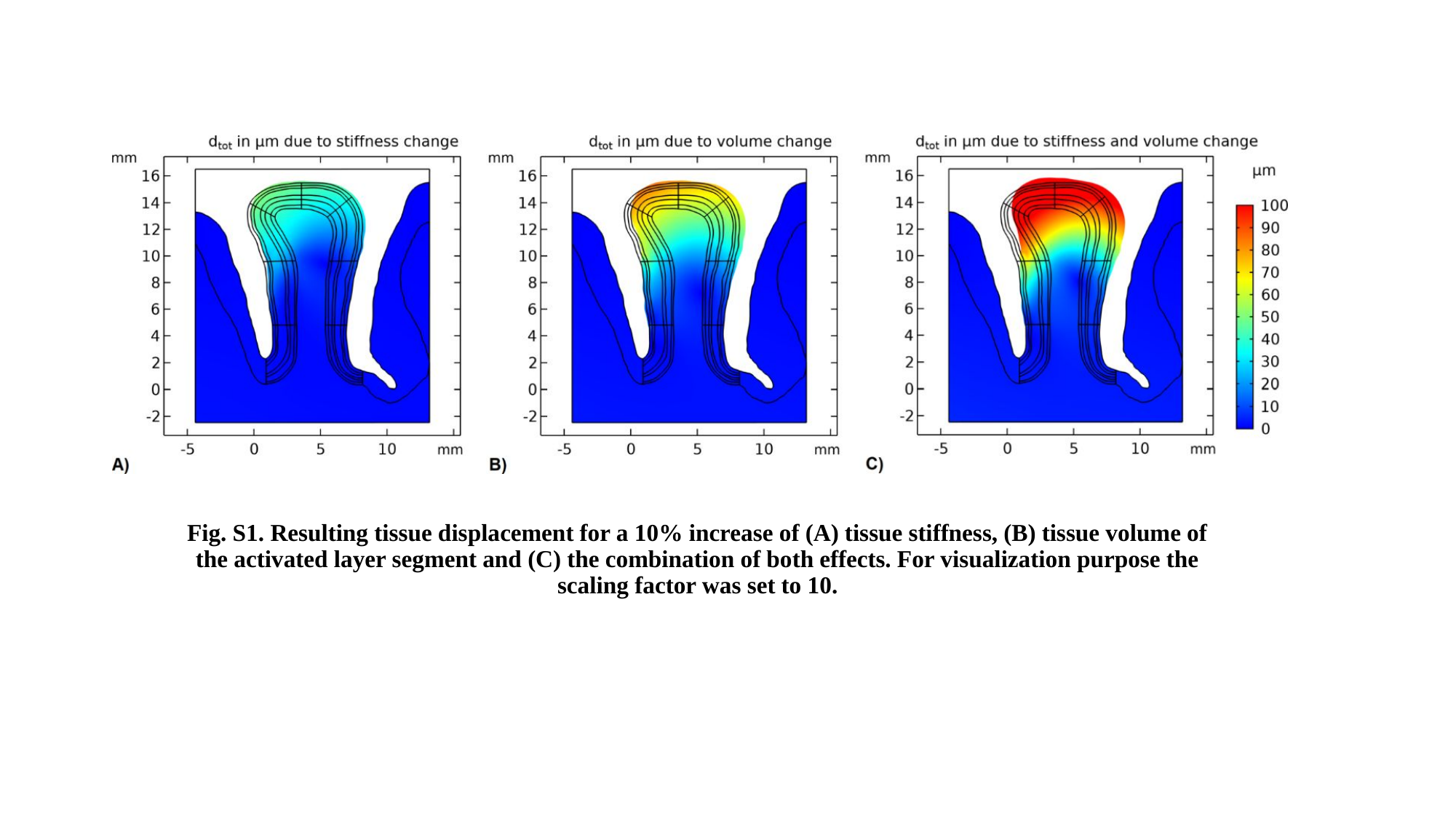

Fig. S1. Resulting tissue displacement for a 10% increase of (A) tissue stiffness, (B) tissue volume of the activated layer segment and (C) the combination of both effects. For visualization purpose the scaling factor was set to 10.

## Slide 2
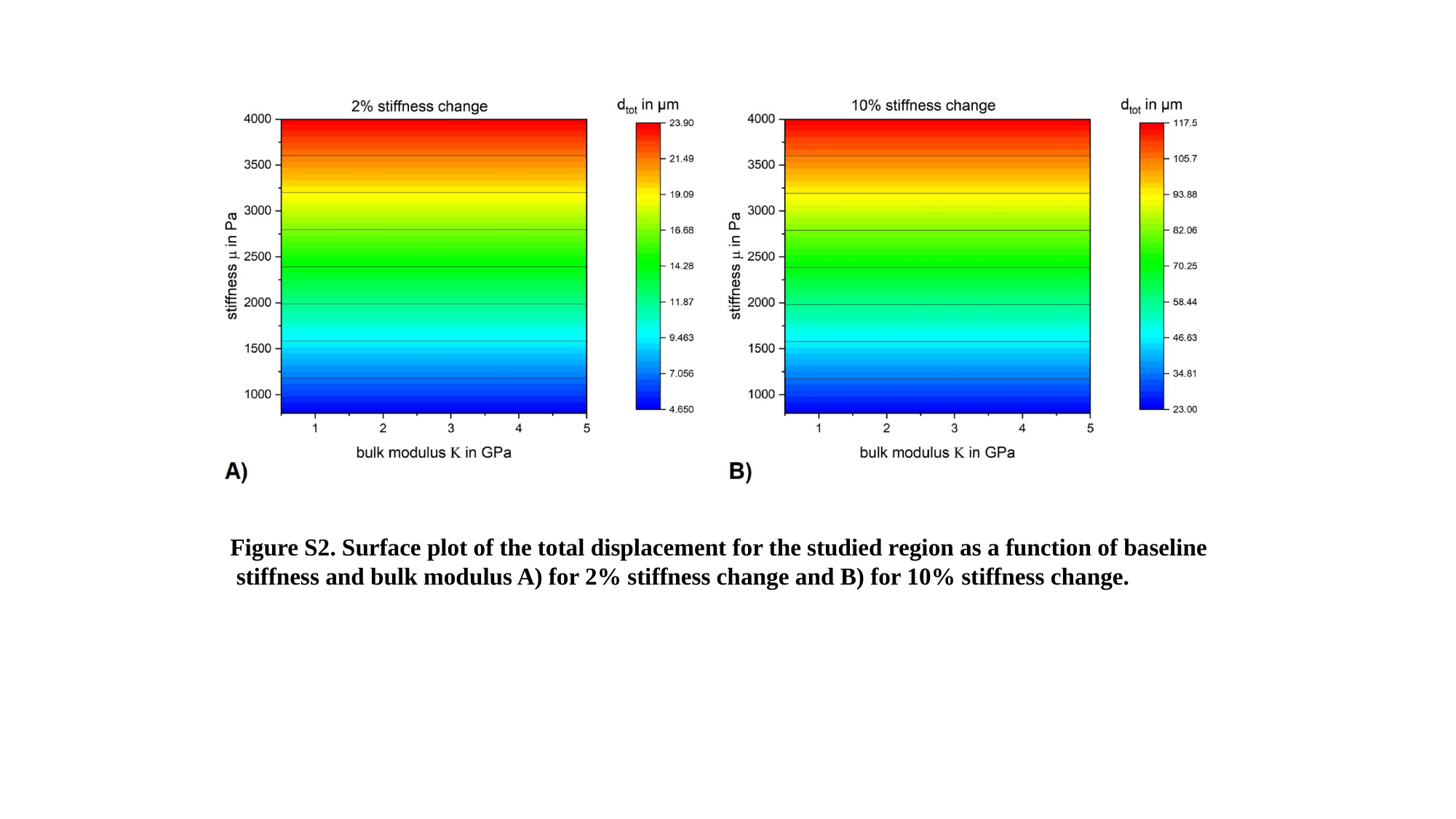

Figure S2. Surface plot of the total displacement for the studied region as a function of baseline
 stiffness and bulk modulus A) for 2% stiffness change and B) for 10% stiffness change.

## Slide 3
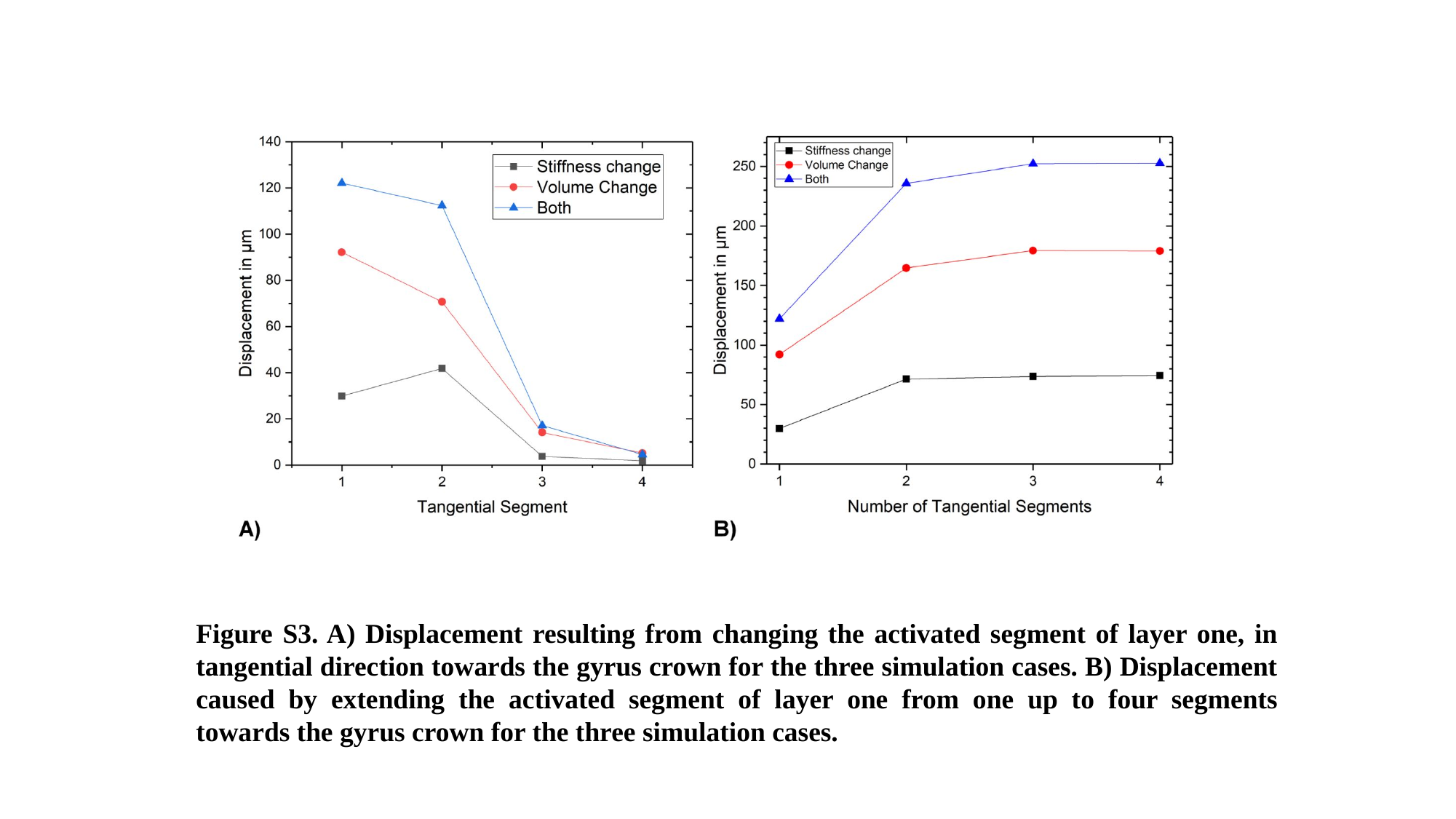

Figure S3. A) Displacement resulting from changing the activated segment of layer one, in tangential direction towards the gyrus crown for the three simulation cases. B) Displacement caused by extending the activated segment of layer one from one up to four segments towards the gyrus crown for the three simulation cases.

## Slide 4
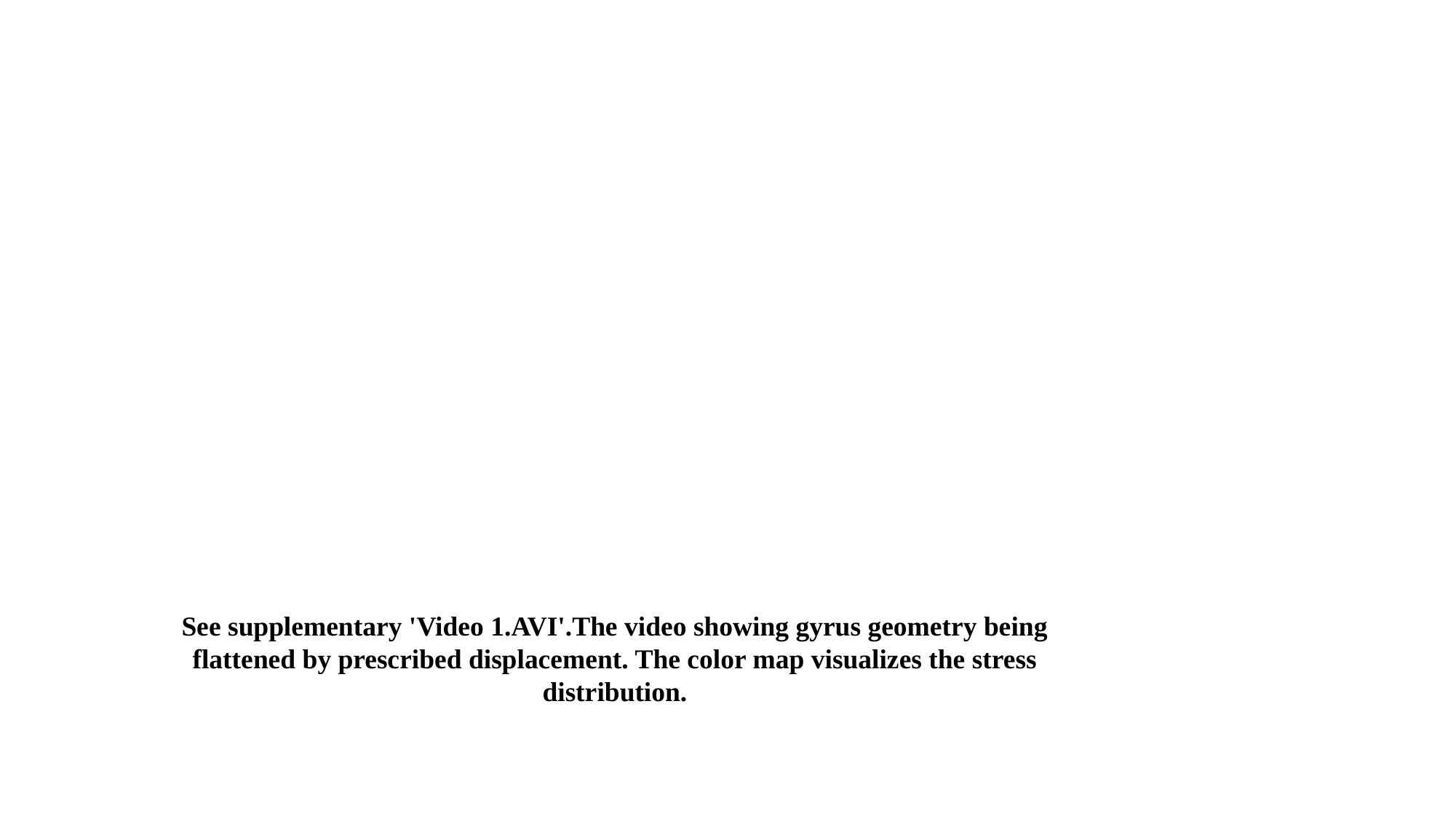

See supplementary 'Video 1.AVI'.The video showing gyrus geometry being flattened by prescribed displacement. The color map visualizes the stress distribution.
